# Mitochondrial genome of *Leocrates chinensis* Kinberg, 1866 (Annelida: Hesionidae)

**DOI:** 10.1080/23802359.2023.2167480

**Published:** 2023-01-25

**Authors:** Xiaolong Li, Deyuan Yang, Jian-Wen Qiu, Penglong Liu, Dehao Meng, Hongmei Zhu, Limei Guo, Site Luo, Zhi Wang, Caihuan Ke

**Affiliations:** a State Key Laboratory of Marine Environmental Science, College of Ocean and Earth Sciences, Xiamen University, Xiamen, China; b National Taiwan Ocean University, Keelung, Taiwan, China; c College of the Environment and Ecology, Xiamen University, Xiamen, China; d Southern Marine Science and Engineering Guangdong Laboratory (Guangzhou), Guangzhou, China; e Department of Biology, Hong Kong Baptist University, Hong Kong, China

**Keywords:** *Leocrates chinensis*, China seas, mitogenome, phylogenetic analysis

## Abstract

We report the complete mitochondrial genome of *Leocrates chinensis* Kinberg, 1866 – the type species of the genus. It is 15061 bp long, and contains 13 protein-coding genes (PCGs), 22 tRNA genes (tRNAs), and 2 rRNA genes (rRNAs), and 1 putative control region. Phylogenetic analysis indicated that *L. chinensis* was placed as sister to *Sirsoe methanicola* (BS = 100) of the same family Hesionidae.

## Introduction

*Leocrates chinensis* Kinberg et al. 1866 is a hesionid widely distributed in southeastern Chinese waters. The holotype of *L. chinensis* was collected from Victoria Harbor, Hong Kong during the Swedish naval frigate *Eugenie’*s around-the-world cruise during 1851–1853, and was later briefly described by Kinberg et al. (1866) as the type species of the newly established hesionid genus *Leocrates*. A recent study redescribed *L. chinensis* with morphological details based on specimens from the type locality (Wang et al. 2018). Nevertheless, since the specimens were preserved in formalin, no attempt was made to extract DNA and determine the placements of this species and Hesionidae in tree of life. Therefore, we conducted genome skimming (Zhang et al. 2018) of *Leocrates chinensis* to help clarify the species status of *Leocrates* specimens that have been identified as *L. chinensi*s based on *cox1* (accession number: OL763897) and several other loci (i.e. 16S, 18S, and 28S, with accession numbers: OL764386, OP104345, and OP104348, respectively) that have been used in species classification of Hesionidae (Rouse et al. 2018; Wang et al. 2020), and to provide resources for studies of mitochondrial genome evolution in the family that currently has only one sequenced mitochondrial genome (*Sirsoe methanicola*, accession no.: OM914591; Lim et al. 2022).

## Materials

**Figure 1.**
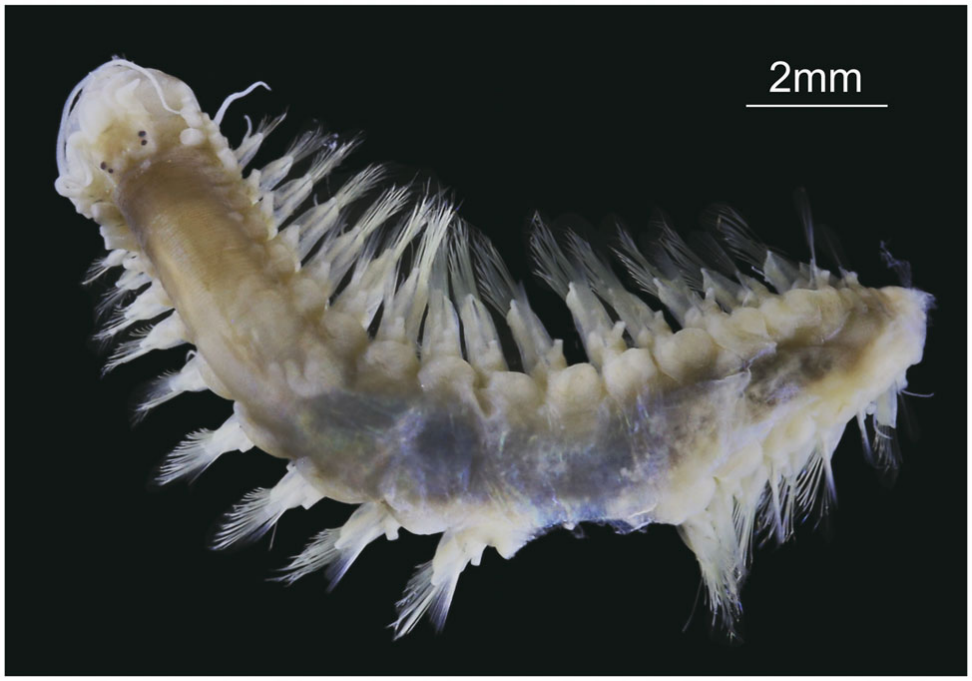
Species reference image of *Leocrates chinensis* (voucher no: XMU-Pol-2021-354). This specimen was collected from subtidal muddy sediment (22°14.160′N, 114°11.115′E, water depth 9.1 m) of the Hong Kong, China on 10 June 2020, and is now deposited in the Biology of Marine Benthic Invertebrates Group, Xiamen University (Photo: Zhi Wang).

The single *Leocrates chinensis* specimen used in this study was collected from the subtidal muddy sediment (Site M(4), 22°14.160′N, 114°11.115′E, water depth 9.1 m) of the Hong Kong, China on 10 June 2020. The specimen was fixed and preserved in 100% ethanol, and both the specimen and its genomic DNA are now deposited in the Biology of Marine Benthic Invertebrates Group, College of Ocean and Earth Sciences, Xiamen University (https://www.xmu.edu.cn/, Zhi Wang, zhiwang00kxy@xmu.edu.cn) under the voucher number XMU-Pol-2021-354 (Figure 1).

## Methods

**Figure 2.**
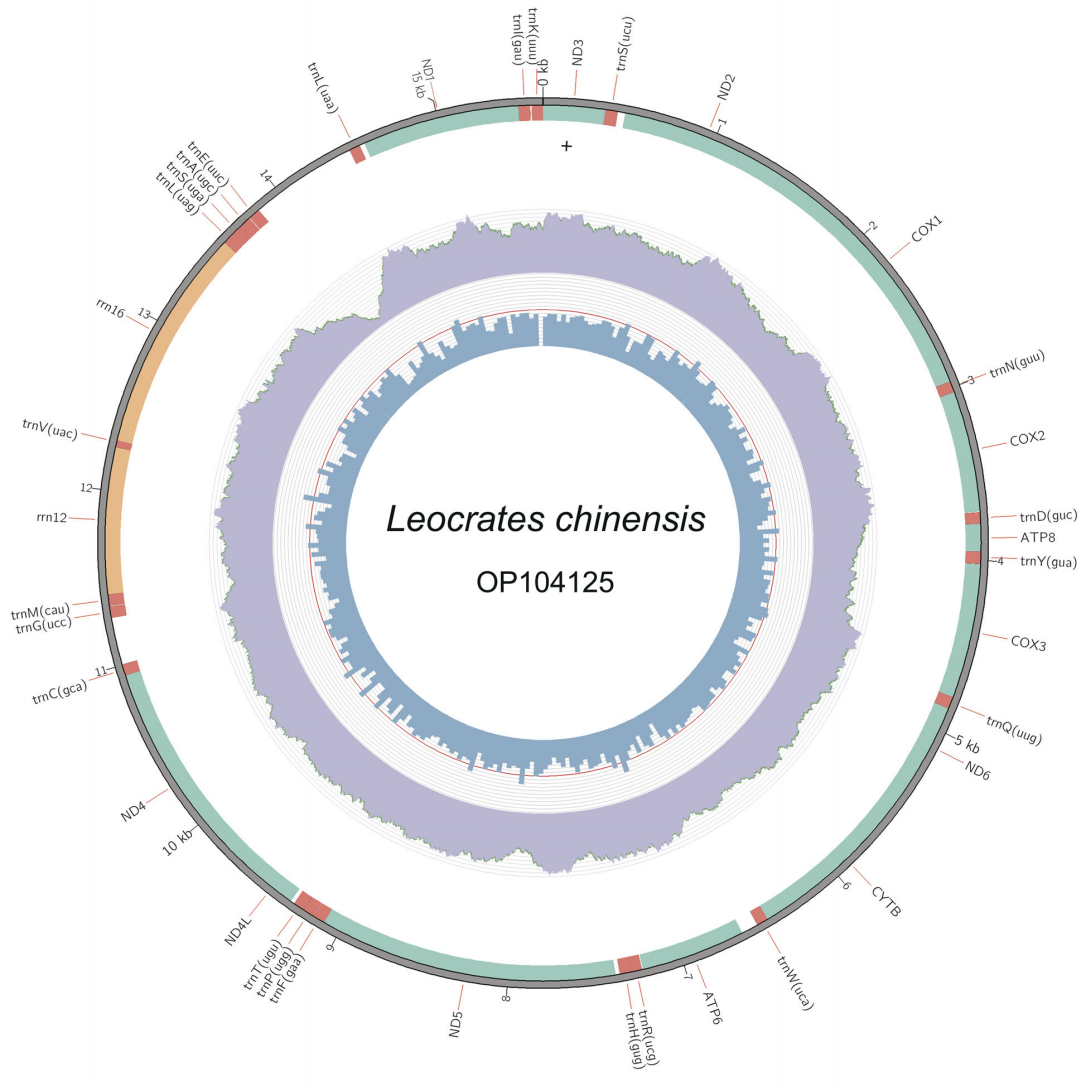
The complete mitogenome of *Leocrates chinensis*. The middle circles and innermost represent depth distribution and GC content, respectively. The outermost circle shows gene arrangements, with green for PCGs fragments, orange for rRNAs and red for tRNAs.

Whole genomic DNA was extracted by using TIANamp Genomic DNA Kit (TIANGEN, Beijing, China). The sequencing library was produced by using the Illumina Truseq™ DNA Sample Preparation Kit (Illumina, San Diego, USA) according to the manufacturer’s recommendations. The prepared library was loaded on the Illumina Novaseq 6000 platform for PE 2 × 150 bp sequencing at Novogene Company (Beijing, China). Sequence quality of raw genomic data was assessed using FastQC v.0.11.5 software (http://www.bioinformatics.babraham.ac.uk/projects/fastqc). Quality trimming and filtering of data was performed using fastp v.0.23.2 (Chen et al. 2018), reads containing more than 5% unknown nucleotides, and low-quality reads (reads containing more than 50% bases with *Q*-value ≤20) and all unpaired reads were discarded. To ensure the consistency in assembly result, the genome assembly was conducted using two methods, i.e. the filtered data were used to assemble the complete mitochondrial genome using the GetOrganelle v.1.7.6.1 pipeline with ‘animal_mt’ in the default database as seed reads (Jin et al. 2020) and NovoPlasty v.4.3.1 with the *cox1* (OL763897) sequence as the seed reads (Dierckxsens et al. 2017). To check the coverage depth of the assembled mitogenome, “samtools depth” command in Samtools v.1.6 (Danecek et al. 2021) was used to calculate the coverage depth, and Circos v.0.69 (Krzywinski et al. 2009) was used to draw the coverage plot (as shown in Figure 2). The annotation of the mitogenome was made with the MITOS2 webserver (Donath et al. 2019) and the Mitoz annotation module (Meng et al. 2019). Additionally, we used maximum-likehood method with the software Phylosuite to reconstruct the phylogenetic tree (Zhang et al. 2020) among Errantia polychaete families. For the three Nereidiformia families, i.e. Hesionidae, Syllidae, and Nereididae, sequences of two genera in each family were included in the analyses; and for the other families including the outgroups (i.e. Capitellidae, Urechidae, Lumbricidae), only one genera was used for each family. All the mitogenomic data included in the analysis came from those published papers (Table 1), and concatenated protein-coding genes (PCGs) of these mitogemones were used in the analysis with each coding gene aligned individually using Mafft v.7.313 (Katoh and Standley 2013) under Codon alignment mode. Ambiguously aligned regions were removed with Gblocks v.0.91 with default settings (Castresana 2000). The best-fitting partition models (GTR + F + I + G4) for ML analysis were selected by the software ModelFinder (Kalyaanamoorthy et al. 2017).

**Table 1.** Mitochondrial sequences used in the phylogenetic analysis as shown in Figure 3.

Family	Species name	Genbank accession numbers	Referenes
Eunicidae	*Marphysa tripectinata*	MG205526	Chen et al. 2019
Onuphidae	*Diopatra cuprea*	MZ434957	Hamilton et al. 2022
Goniadidae	*Goniada japonica*	KP867019	Chen et al. 2016
Glyceridae	*Glycera capitata*	KT989319	Richter et al. 2015
Hesionidae	*Sirsoe methanicola*	OM914591	Lim et al. 2022
**Hesionidae**	***Leocrates chinensis***	**OP104125**	**This study**
Nereididae	*Alitta succinea*	MN812981	Alves et al. 2020
Nereididae	*Platynereis dumerilii*	AF178678	Boore and Brown 2000
Syllidae	*Trypanobia cryptica*	KR534503	Aguado et al. 2015
Syllidae	*Ramisyllis multicaudata*	KR534502	Aguado et al. 2015
Nephtyidae	*Nephtys* sp.	EU293739	Vallès et al. 2008
Eulepethidae	*Eulepethus nanhaiensis*	KY753834	Zhang et al. 2018
Aphroditidae	*Aphrodita australis*	MN334532	Wang et al. 2019
Acoetidae	*Panthalis oerstedi*	KY753832	Zhang et al. 2018
Polynoidae	*Halosydna* sp.	KY753830	Zhang et al. 2018
Iphionidae	*Iphione* sp.	KY753835	Zhang et al. 2018
Amphinomidae	*Eurythoe complanata*	KT726962	Weigert et al. 2016
Orbiniidae	*Orbinia latreillii*	AY961084	Bleidorn et al. 2006
Capitellidae	*Notomastus* sp.	LC661358	Kobayashi et al. 2022
Urechidae	*Urechis unicinctus*	EF656365	Wu et al. 2009
Lumbricidae	*Lumbricus rubellus*	MN102127	Zhang et al. 2019

## Results

**Figure 3.**
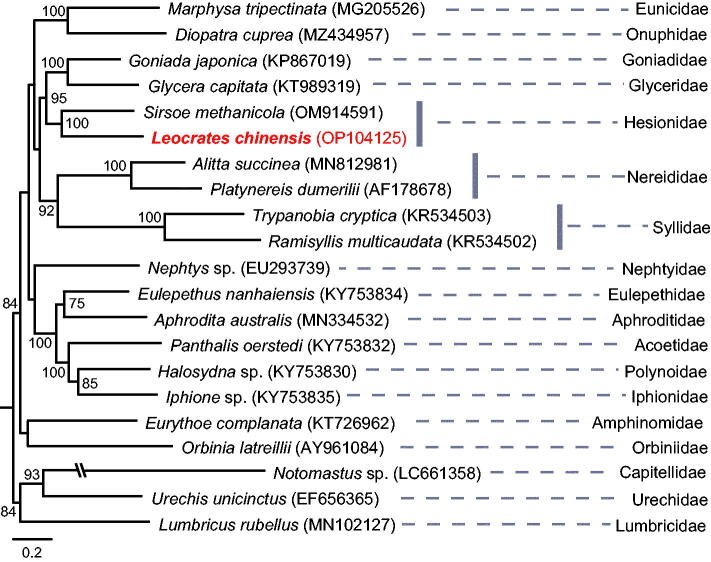
Phylogenetic tree reconstructed with Maximum Likelihood (ML) method. The tree was constructed based on concatenated nucleotide sequences of 13 protein-coding genes (PCGs) of 20 polychaetous species in 17 families and 1 clitellate species. Values of robustness were calculated from ML analyses, and only bootstrap (BS) values ≥50 are shown at nodes. GenBank accession numbers used are listed after the species names. The scale bar indicates the number of substitutions per site.

The two assembly pipelines (i.e. GetOrganelle and NovoPlasty) gave an exact identical circular mitogenome. The size of the complete mitochondrial genome was 15,061 bp, and the data were submitted to the NCBI (accession no.: OP104125). The genome consisted of 60.3% A + T, with 28.8% of A, 31.5% of T, 14.8% of G, and 24.9% of C. The genome contains 13 PCGs, i.e. 2 rRNA genes, 22 tRNA genes, and 1 putative control region consisting of 620 bp (Figure 2). Six PCGs started with ATG codon, two PCGs started with ATT codon, one PCG started with ATC and four PCGs started with ATA. All 13 PCGs terminated with TAA stop codon except *nad4* and *nad2*, which with T stop codon. The real phylogenetic relationships among available Errantia polychaetous families are exhibited in Figure 3. Results showed that *L. chinensis* was sister taxon of *Sirsoe methanicola* (BS = 100), which was the only one sequenced mitochondrial genome in the same family Hesionidae.

## Discussion and conclusion

The first complete mitochondrial genome of the polychaetous species *Leocrates chinensis* is reported in this study, which is also the first mitogenome reported in the genus *Leocrates.* We used two different assembly methods (i.e. GetOrganelle v.1.7.6.1 and NovoPlasty v.4.3.1) to ensure the consistency of the assembly result. We noted that there was slight negative coverage anomaly in the control region (CR). It might be due to compositional bias affecting sequencing. As we all know, the control region has high A + T content, and this region always facing sequencing problems. Based on our experiences in mitogenomic studies of polychaetes, the CR is difficult to assemble and it always has a slight negative coverage anomaly. This study provides a valuable data to studying the phylogenetic and evolutionary history of annelid polychaetes. And to make better understanding of the phylogeny within Hesionidae, mitogenomic studies on more genera of this family, such as *Oxydromus*, *Podarkeopsis*, *Micropodarke*, etc., should be put into agenda in future studies.

## Data Availability

The genome sequence data that support the findings of this study are openly available in GenBank of NCBI at [https://www.ncbi.nlm.nih.gov](https://www.ncbi.nlm.nih.gov/) under the accession no. OP104125. The associated BioProject, Bio-Sample, and SRA numbers are PRJNA859499, SAMN29785536, and SRR20276768, respectively.
